# Supplementary material for: General recipe to realize photonic-crystal surface-emitting lasers with 100-W-to-1-kW single-mode operation
Source: Nat Commun. 2022 Jul 4;13:3262. doi: 10.1038/s41467-022-30910-7 (PMC9253024; doi:10.1038/s41467-022-30910-7)
Supplement: Supplementary file 1 — Supplementary Information [file 41467_2022_30910_MOESM1_ESM.pdf]

SUPPLEMENTARY INFORMATION

General recipe to realize photonic-crystal surface-emitting lasers with 100-W-to-1-kW single-mode operation

Takuya Inoue[1,3,*], Masahiro Yoshida[2,3], John Gelleta[2,3], Koki Izumi[2], Keisuke Yoshida[2], Kenji Ishizaki[2], Menaka De Zoysa[1], and Susumu Noda[1,2,*]

[1] Photonics and Electronics Science and Engineering Center, Kyoto University,

[2] Department of Electronic Science and Engineering, Kyoto University,

Kyoto 615-8510, Japan

[3]These authors contributed equally to this work.

*To whom correspondence should be addressed.

E-mail: t_inoue@qoe.kuee.kyoto-u.ac.jp, snoda@kuee.kyoto-u.ac.jp

1. Details of coupling coefficients of photonic crystals

The coupled-wave matrix $\mathbf{C}_{\text{Hermitian}}+\mathbf{C}_{\text{non-Hermitian}}$ can be written as the sum of three matrices:

$$\mathbf{C}_{\text{Hermitian}}+\mathbf{C}_{\text{non-Hermitian}}=\mathbf{C}_{\text{1D}}+\mathbf{C}_{\text{rad}}+\mathbf{C}_{\text{2D}}, \tag{S1}$$

$$\mathbf{C}_{\text{1D}}=\begin{pmatrix} 0 & -\kappa_{2,0} & 0 & 0 \\ -\kappa_{-2,0} & 0 & 0 & 0 \\ 0 & 0 & 0 & -\kappa_{0,2} \\ 0 & 0 & -\kappa_{0,-2} & 0 \end{pmatrix}, \tag{S2}$$

$$\mathbf{C}_{\text{rad}}=\begin{pmatrix} \zeta_{1,0}^{(1,0)} & -\zeta_{1,0}^{(-1,0)} & 0 & 0 \\ -\zeta_{-1,0}^{(1,0)} & \zeta_{-1,0}^{(-1,0)} & 0 & 0 \\ 0 & 0 & \zeta_{0,1}^{(0,1)} & -\zeta_{0,1}^{(0,-1)} \\ 0 & 0 & -\zeta_{0,-1}^{(0,1)} & \zeta_{0,-1}^{(0,-1)} \end{pmatrix}, \tag{S3}$$

$$\mathbf{C}_{\text{2D}}=\begin{pmatrix} \chi_{y,1,0}^{(1,0)} & -\chi_{y,1,0}^{(-1,0)} & -\chi_{y,1,0}^{(0,1)} & \chi_{y,1,0}^{(0,-1)} \\ -\chi_{y,-1,0}^{(1,0)} & \chi_{y,-1,0}^{(-1,0)} & \chi_{y,-1,0}^{(0,1)} & -\chi_{y,-1,0}^{(0,-1)} \\ -\chi_{x,0,1}^{(1,0)} & \chi_{x,0,1}^{(-1,0)} & \chi_{x,0,1}^{(0,1)} & -\chi_{x,0,1}^{(0,-1)} \\ \chi_{x,0,-1}^{(1,0)} & -\chi_{x,0,-1}^{(-1,0)} & -\chi_{x,0,-1}^{(0,1)} & \chi_{x,0,-1}^{(0,-1)} \end{pmatrix}, \tag{S4}$$

where

$$\kappa_{i,j}=-\frac{\omega_0^2}{2\beta_0 c^2}\int \xi_{i,j}\left|\Theta_0(z)\right|^2 dz\,, \tag{S5}$$

$$\zeta_{p,q}^{(r,s)}=-\frac{\omega_0^4}{2\beta_0 c^4}\iint \xi_{p,q}\xi_{-r,-s}G\left(z,z'\right)\Theta_0(z')\Theta_0^*(z)dz'dz\,, \tag{S6}$$

$$\chi_{j,p,q}^{(r,s)}=-\frac{\omega_0^2}{2\beta_0 c^2}\sum_{\sqrt{m^2+n^2}>1}\xi_{p-m,q-n}\varsigma_{j,m,n}^{(r,s)},\quad j=x,y\,. \tag{S7}$$

$$\xi_{m,n}=\frac{1}{a^2}\int_{\text{unitcell}} n_{\text{pc}}^2(\mathbf{r})e^{i(m\beta_0 x+n\beta_0 y)}dxdy\,. \tag{S8}$$

The derivation of these coupled-wave matrix is detailed in our previous papers [S1,S2]. It should be noted that the minus signs of the coupling coefficients in Eqs. (S2), (S3), (S4)

depend on the definition of the direction of the electric fields (in this paper, we define the electric fields of the fundamental waves as $\mathbf{E}_{1,0}= R_x\mathbf{e}_y$, $\mathbf{E}_{-1,0}=S_x(-\mathbf{e}_y)$, $\mathbf{E}_{0,1}= R_y(-\mathbf{e}_x)$, $\mathbf{E}_{0,-1}= S_y\mathbf{e}_x$, where $\mathbf{e}_x$ and $\mathbf{e}_y$ are unit vectors in x and y directions, respectively). $\Theta_0(z)$ denotes the vertical profile of the electric field, $G(z,z')$ denotes the Green's function for a point source at z=z', and $n_{\mathrm{pc}}^2(\mathbf{r})$ denotes the in-plane refractive index distribution of the photonic crystal layers. $\mathbf{C}_{\mathrm{1D}}$, $\mathbf{C}_{\mathrm{rad}}$, and $\mathbf{C}_{\mathrm{2D}}$ correspond to 1D back-diffraction coupling, 1D coupling via out-of-plane radiative waves, and 2D coupling via high-order waves, respectively. $\mathbf{C}_{\mathrm{1D}}$ and $\mathbf{C}_{\mathrm{2D}}$ are Hermitian matrices, while $\mathbf{C}_{\mathrm{rad}}$ contains both Hermitian and non-Hermitian components. Eqs. (S2)–(S4) become equivalent to Eqs. (3) and (4) when we re-define the coupling coefficients as follows;

$$\kappa_{\mathrm{1D}} = -\kappa_{2,0} - \chi_{y,1,0}^{(-1,0)} + \mathrm{Re}\left(\zeta_{1,0}^{(1,0)}\right)e^{i\theta_{\mathrm{pc}}}, \tag{S9}$$

$$\kappa_{\mathrm{2D+}} = -\chi_{y,1,0}^{(0,1)}, \tag{S10}$$

$$\kappa_{\mathrm{2D-}} = \chi_{y,1,0}^{(0,-1)}, \tag{S11}$$

$$\kappa_{11} = \chi_{y,1,0}^{(1,0)} + \mathrm{Re}\left(\zeta_{1,0}^{(1,0)}\right), \tag{S12}$$

$$\mu = \mathrm{Im}\left(\zeta_{1,0}^{(1,0)}\right), \tag{S13}$$

$$\theta_{\mathrm{pc}} = \pi + \arg\left(\zeta_{1,0}^{(-1,0)}\right) - \arg\left(\zeta_{1,0}^{(1,0)}\right) = \pi + 2\arg\left(\xi_{1,0}\right). \tag{S14}$$

In Eq. (S14), θ_{pc} represents the phase of the non-Hermitian 180°-coupling (for example, S_x ⇒ radiative waves ⇒ R_x). θ_{pc} is determined by the position at which non-Hermitian couplings occur relative to the origin of the coordinate system (x=0, fixed at the center of

the unit cell). For example, in a simple single-lattice photonic crystal whose circular air hole is placed at the center of the unit cell (x=0) (Fig. S1a), the position at which non-Hermitian couplings occur via a radiative wave (x_r) is exactly located at x=0. In this case, the phase change in the ±180°-coupling θ_{pc} is equal to π because of our presupposition that the direction of the electric field vector is opposite for R_x and S_x. On the other hand, in a double-lattice photonic crystal shown in Fig. S1b, the situation is different: when the centroids of the air holes are equidistant from the center of the unit cell (x=0), the position at which the non-Hermitian couplings occur (x_r) deviates from x=0. This is because the position $x=x_r$ is mainly determined by the center of gravity of the refractive index distribution, which is asymmetric with respect to the origin (x=0) in the double-lattice photonic crystal. Accordingly, the phase change of the ±180°-coupling θ_{pc} becomes $2\beta x_r+\pi$ as shown in Fig. S1b, where $\beta=2\pi/a$. In our numerical analysis, θ_{pc} is almost constant, at 0.92π, because the variance of the hole parameters (d and $2x$) are no larger than a few nanometers.

Since the value of θ_{pc} is dependent on the position of the air holes, its value changes following arbitrary global translations of the air holes as shown in Fig. S1c. In the main text, we consider $(\kappa_{1D}+\kappa_{2D-})e^{-i\theta_{pc}}$ as a phase-invariant effective Hermitian coupling coefficient, since this quantity signifies the relative phase difference between Hermitian ±180°-couplings and non-Hermitian ±180°-couplings and is invariant to global

translations of the lattice points along y=x.

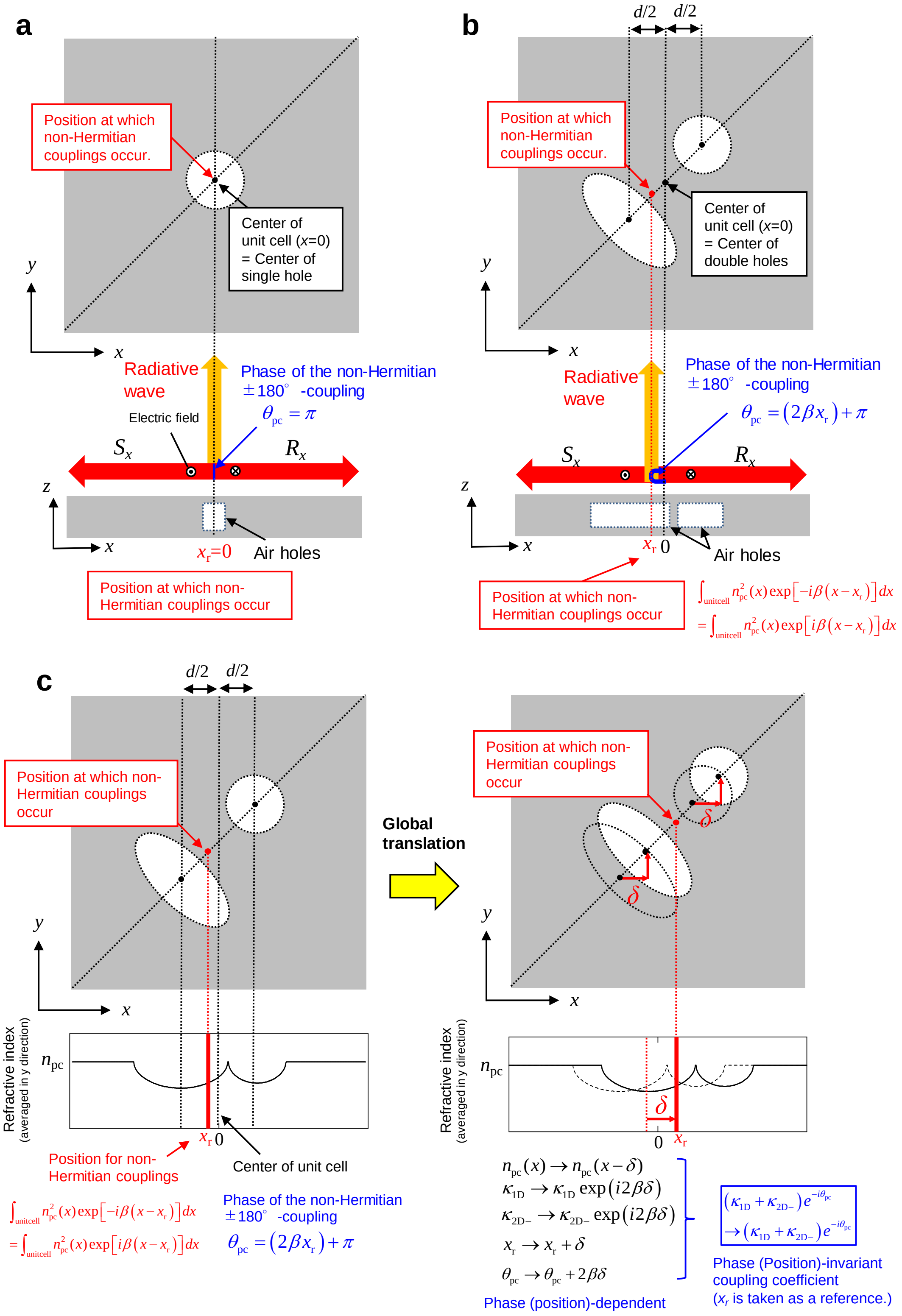

Fig. S1| Physical meanings of θ_{pc}. a, Schematic illustration of θ_{pc} in a single-lattice

photonic crystal. **b.** Schematic illustration of θ_{pc} in a double-lattice photonic crystal. **c.** Phase change of coupling coefficients following a global translation of the air holes along $y=x$.

2. Derivation of eigenfrequencies

From Eqs. (2)–(5), we obtain

$$\begin{aligned}\mathbf{C} &= \mathbf{C}_{\text{Hermitian}} + \mathbf{C}_{\text{non-Hermitian}} + \mathbf{C}_{\text{non-Gamma}} \\ &= \begin{pmatrix} \kappa_{11}+i\mu & \kappa_{1\mathrm{D}}+i\mu e^{i\theta_{\mathrm{pc}}} & \kappa_{2\mathrm{D}+} & \kappa_{2\mathrm{D}-} \\ \kappa_{1\mathrm{D}}^{*}+i\mu e^{-i\theta_{\mathrm{pc}}} & \kappa_{11}+i\mu & \kappa_{2\mathrm{D}-}^{*} & \kappa_{2\mathrm{D}+} \\ \kappa_{2\mathrm{D}+} & \kappa_{2\mathrm{D}-} & \kappa_{11}+i\mu & \kappa_{1\mathrm{D}}+i\mu e^{i\theta_{\mathrm{pc}}} \\ \kappa_{2\mathrm{D}-}^{*} & \kappa_{2\mathrm{D}+} & \kappa_{1\mathrm{D}}^{*}+i\mu e^{-i\theta_{\mathrm{pc}}} & \kappa_{11}+i\mu \end{pmatrix} + \begin{pmatrix} k_x & 0 & 0 & 0 \\ 0 & -k_x & 0 & 0 \\ 0 & 0 & k_y & 0 \\ 0 & 0 & 0 & -k_y \end{pmatrix}.\end{aligned} \tag{S15}$$

Here, we assume that the deviation from the Γ-point ($|k_x|$, $|k_y|$) is much smaller than the reciprocal wavenumber β_0. Consider the following matrix for basis transformation:

$$P = \frac{1}{\sqrt{2}}\begin{pmatrix} 1 & 0 & 1 & 0 \\ 0 & 1 & 0 & 1 \\ 1 & 0 & -1 & 0 \\ 0 & 1 & 0 & -1 \end{pmatrix}. \tag{S16}$$

Block diagonalization of Eq. (S15) can be performed as follows;

$$\begin{aligned}\mathbf{P}^{-1}\mathbf{C}\mathbf{P} &= \begin{pmatrix} \kappa_{11}+\kappa_{2\mathrm{D}+}+i\mu & \kappa_{1\mathrm{D}}+\kappa_{2\mathrm{D}-}+i\mu e^{i\theta_{\mathrm{pc}}} & 0 & 0 \\ \kappa_{1\mathrm{D}}^{*}+\kappa_{2\mathrm{D}-}^{*}+i\mu e^{-i\theta_{\mathrm{pc}}} & \kappa_{11}+\kappa_{2\mathrm{D}+}+i\mu & 0 & 0 \\ 0 & 0 & \kappa_{11}-\kappa_{2\mathrm{D}+}+i\mu & \kappa_{1\mathrm{D}}-\kappa_{2\mathrm{D}-}+i\mu e^{i\theta_{\mathrm{pc}}} \\ 0 & 0 & \kappa_{1\mathrm{D}}^{*}-\kappa_{2\mathrm{D}-}^{*}+i\mu e^{-i\theta_{\mathrm{pc}}} & \kappa_{11}-\kappa_{2\mathrm{D}+}+i\mu \end{pmatrix} + \begin{pmatrix} k_+ & 0 & k_- & 0 \\ 0 & -k_+ & 0 & -k_- \\ k_- & 0 & k_+ & 0 \\ 0 & -k_- & 0 & -k_+ \end{pmatrix} \\ &= \begin{pmatrix} \kappa_{11}+\kappa_{2\mathrm{D}+}+i\mu & \left[(\kappa_{1\mathrm{D}}+\kappa_{2\mathrm{D}-})e^{-i\theta_{\mathrm{pc}}}+i\mu\right]e^{i\theta_{\mathrm{pc}}} & 0 & 0 \\ \left[\left\{(\kappa_{1\mathrm{D}}+\kappa_{2\mathrm{D}-})e^{-i\theta_{\mathrm{pc}}}\right\}^{*}+i\mu\right]e^{-i\theta_{\mathrm{pc}}} & \kappa_{11}+\kappa_{2\mathrm{D}+}+i\mu & 0 & 0 \\ 0 & 0 & \kappa_{11}-\kappa_{2\mathrm{D}+}+i\mu & \left[(\kappa_{1\mathrm{D}}-\kappa_{2\mathrm{D}-})e^{-i\theta_{\mathrm{pc}}}+i\mu\right]e^{i\theta_{\mathrm{pc}}} \\ 0 & 0 & \left[\left\{(\kappa_{1\mathrm{D}}-\kappa_{2\mathrm{D}-})e^{-i\theta_{\mathrm{pc}}}\right\}^{*}+i\mu\right]e^{-i\theta_{\mathrm{pc}}} & \kappa_{11}-\kappa_{2\mathrm{D}+}+i\mu \end{pmatrix} \\ &\quad + \begin{pmatrix} k_+ & 0 & k_- & 0 \\ 0 & -k_+ & 0 & -k_- \\ k_- & 0 & k_+ & 0 \\ 0 & -k_- & 0 & -k_+ \end{pmatrix}\end{aligned} \tag{S17}$$

where

$$k_+ = \frac{1}{2}\left(k_x + k_y\right),\quad k_- = \frac{1}{2}\left(k_x - k_y\right). \tag{S18}$$

In Eq. (S17), the upper (lower) 2×2 block corresponds to modes A and C (B and D). The complex eigenfrequencies at the Γ point [Eqs. (9) and (10)] can be directly obtained from the first term of the right hand side of Eq. (S17). The eigenfrequencies of the modes in the Γ-M direction (k_x=k_y) can be also analytically solved, which gives the result shown in Eq. (16). When we consider the modes in the Γ-M' direction (k_x=−k_y), the coupled-wave matrix in Eq. (S17) cannot be diagonalized in a simple form. It should be noted, however, that the complex eigenfrequencies in the Γ-M' direction and those in the Γ-M direction are similar in the most cases owing to the following relationship:

$$\det\left(\mathbf{P}^{-1}\mathbf{C}\mathbf{P} - \lambda\mathbf{I}\right)\Big|_{k_x=-k_y=\Delta k/\sqrt{2}} = \det\left(\mathbf{P}^{-1}\mathbf{C}\mathbf{P} - \lambda\mathbf{I}\right)\Big|_{k_x=k_y=\Delta k/\sqrt{2}} + 2\left(\Delta k\right)^2\left(\left|\kappa_{2\mathrm{D}+}\right|^2 - \left|\kappa_{2\mathrm{D}-}\right|^2\right), \tag{S19}$$

where $\mathbf{I}$ denotes a 4×4 identity matrix. According to this equation, the complex eigenfrequencies in the Γ-M' direction and those in the Γ-M direction are identical when the magnitudes of $\kappa_{2\mathrm{D}+}$ and $\kappa_{2\mathrm{D}-}$ are equal, and they are similar in the vicinity of the Γ-point even when the magnitudes of $\kappa_{2\mathrm{D}+}$ and $\kappa_{2\mathrm{D}-}$ are slightly different.

3. Radiation constants of band-edge modes in a C_2-asymmetric photonic crystal

As explained in the main text, when we consider a lattice-point design that completely breaks C_2 symmetry, all of the band-edge modes in Eqs. (9) and (10) in the main text have

non-zero radiation constants. In the specific case of $|\kappa_{1D}+\kappa_{2D-}|\sim 0$, the radiation constants of the four modes are expressed as follows [Eq. (11) in the main text];

$$\begin{aligned}
&\alpha_{A}\sim 0\\
&\alpha_{C}\sim 4\mu \gg 0\\
&\alpha_{B}\sim 2\mu-2\left|\mathrm{Im}\sqrt{\left(2\kappa_{1D}+i\mu e^{i\theta_{pc}}\right)\left(2\kappa_{1D}^{*}+i\mu e^{-i\theta_{pc}}\right)}\right|>0\\
&\alpha_{D}\sim 2\mu+2\left|\mathrm{Im}\sqrt{\left(2\kappa_{1D}+i\mu e^{i\theta_{pc}}\right)\left(2\kappa_{1D}^{*}+i\mu e^{-i\theta_{pc}}\right)}\right|\gg 0
\end{aligned} \quad \text{(S20)}$$

In one exemplary simulation, we change the complex coupling coefficient $\kappa_{1D}+\kappa_{2D-}$(or κ_{1D}) and fix the other parameters as follows: μ=90 cm^{-1}, κ_{2D-} = −70−70i cm^{-1}, κ_{2D+} = −100 cm^{-1}, θ_{pc} = 0.92π. The calculated radiation constants of modes A and B and their difference are shown in Figs. S2a, S2b, and S2c, respectively (it should be noted that the radiation constants of modes C and D are higher than 2μ =180 cm^{-1}). As shown in Fig. S2c, the radiation constant of mode B is at least 20 cm^{-1} higher than that of mode A for almost all of the $\kappa_{1D}+\kappa_{2D-}$ values, which enables single-mode lasing at mode A.

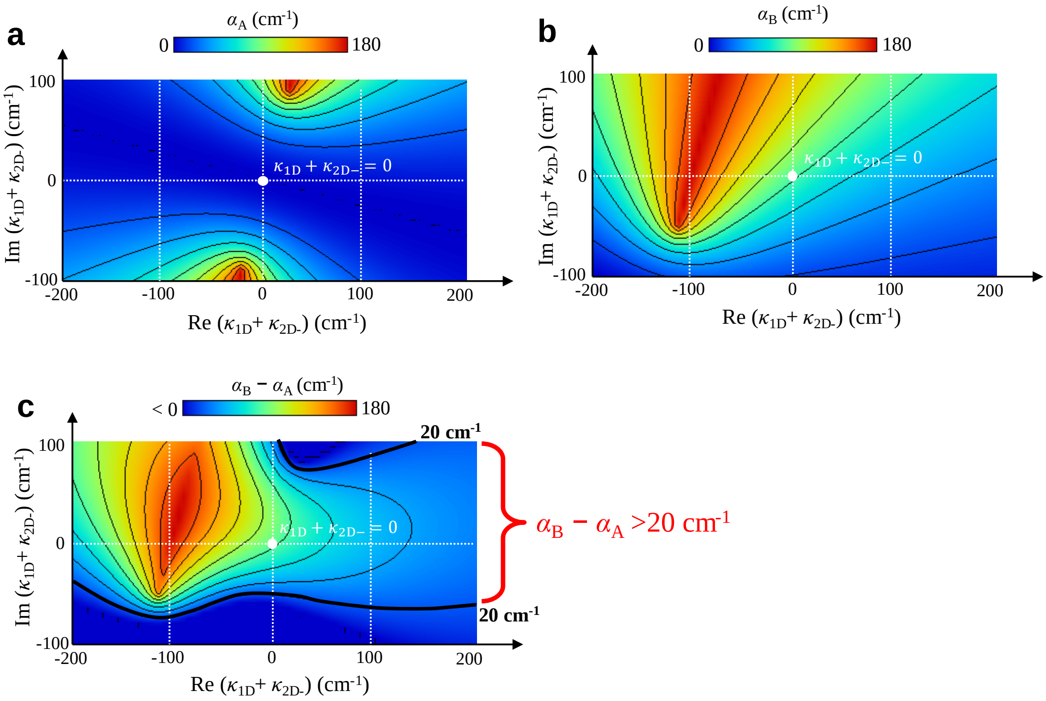

Figure S2| Radiation constants of modes A and B as a function of Hermitian coupling coefficient. a, Mode A. **b,** Mode B. **c,** Difference between mode A and mode B. The interval of the contour lines is 20 cm^{-1} in each figure.

4. Physical origin of radiation from photonic crystals and the role of the imaginary part *I* of the effective Hermitian coupling coefficient

To understand the physical origin of radiation from photonic crystals, we first review the role of the non-Hermitian coupling coefficient ($i\mu$) in the radiation process. Physically, $i\mu$ represents the vertical emission loss that accompanies each of the four fundamental waves as it couples to itself via radiative waves. This coupling coefficient $i\mu$ induces the radiation constant of 2μ for each fundamental wave (R_x, R_y, S_x, S_y) when the mutual coupling between them is ignored. For example, the radiation constant of each

band-edge mode (A, B, C, D) becomes almost equal to 2μ when the in-plane k-vectors ($\mathbf{k}_{//}$) deviate far enough from the Γ point, where the effect of mutual couplings among the four fundamental waves becomes very weak.

On the other hand, at the Γ point of each band, the mutual couplings among the four fundamental waves determine the overall behavior of radiation from the four fundamental waves. As we have explained in Fig. 2 in the main text, the effective Hermitian couplings between the two anti-symmetric electric-field pairs ($R_x + R_y$ and $S_x + S_y$) in modes A and C are determined by [$\kappa_{\mathrm{1D}} + \kappa_{\mathrm{2D-}}, (\kappa_{\mathrm{1D}} + \kappa_{\mathrm{2D-}})^*$], and there are also non-Hermitian ±180°-couplings [$i\mu\exp(\pm i\theta_{\mathrm{pc}})$] between these pairs. Combining these two effects, the coupling matrix for modes A and C is derived as follows:

$$\begin{aligned}(\delta + i\alpha/2)\begin{pmatrix} R_x + R_y \\ S_x + S_y \end{pmatrix} &= \begin{pmatrix} \kappa_{11} + \kappa_{\mathrm{2D+}} + i\mu & \kappa_{\mathrm{1D}} + \kappa_{\mathrm{2D-}} + i\mu e^{i\theta_{\mathrm{pc}}} \\ \kappa_{\mathrm{1D}}^* + \kappa_{\mathrm{2D-}}^* + i\mu e^{-i\theta_{\mathrm{pc}}} & \kappa_{11} + \kappa_{\mathrm{2D+}} + i\mu \end{pmatrix}\begin{pmatrix} R_x + R_y \\ S_x + S_y \end{pmatrix} \\ &= \begin{pmatrix} \kappa_{11} + \kappa_{\mathrm{2D+}} + i\mu & [R + iI + i\mu]e^{i\theta_{\mathrm{pc}}} \\ [R - iI + i\mu]e^{-i\theta_{\mathrm{pc}}} & \kappa_{11} + \kappa_{\mathrm{2D+}} + i\mu \end{pmatrix}\begin{pmatrix} R_x + R_y \\ S_x + S_y \end{pmatrix}\end{aligned} \quad \text{(S21)}$$

From this equation, one can easily understand that the coupling strengths from $S_x + S_y$ to $R_x + R_y$ and vice versa are different when the imaginary part of the phase-invariant effective Hermitian coupling coefficients (I) takes a non-zero value. This asymmetry in the coupling strengths gives rise to the imperfect overall cancellation of radiation for mode A. When the condition of $|I| \ll |R+i\mu|$ is satisfied, the coupling strengths from $S_x + S_y$ to $R_x + R_y$ and vice versa are almost equal, which leads to a

relatively small radiation constant of mode A (<20~30cm^{-1}). This enables lasing with a low-to-moderate threshold current, which is desirable in a practical laser.

To visually understand the physical meaning of the imaginary part of the effective Hermitian coupling coefficient (I), we calculated the electric field distributions of mode A in typical double-lattice photonic crystals with different values of I (corresponding to different values of $2x$ in Fig. 5b). The results of these calculations are shown in Fig. S3, where mode A has a node (shown in blue) around which the electric field vectors circulate. The position of this node is determined by solving the above Eq. (S21), where the vertical radiation is cancelled out. When the value of $|I|$ is almost zero (middle panel), the position of this node coincides with the position for non-Hermitian couplings (x_r) explained in Fig. S1 (shown in red), leading to complete cancellation of the radiation from the two holes. On the other hand, when the value of $|I|$ is larger than zero (left and right panels), the position of the node shifts from x_r, leading to incomplete cancellation of the radiation and a corresponding increase of the radiation constant. Thus, the imaginary part of the phase-invariant effective Hermitian coupling coefficient (I) determines the degree of cancellation of the vertical radiation at the Γ point, which is based on the deviation of the node of the electric field from the position for non-Hermitian couplings.

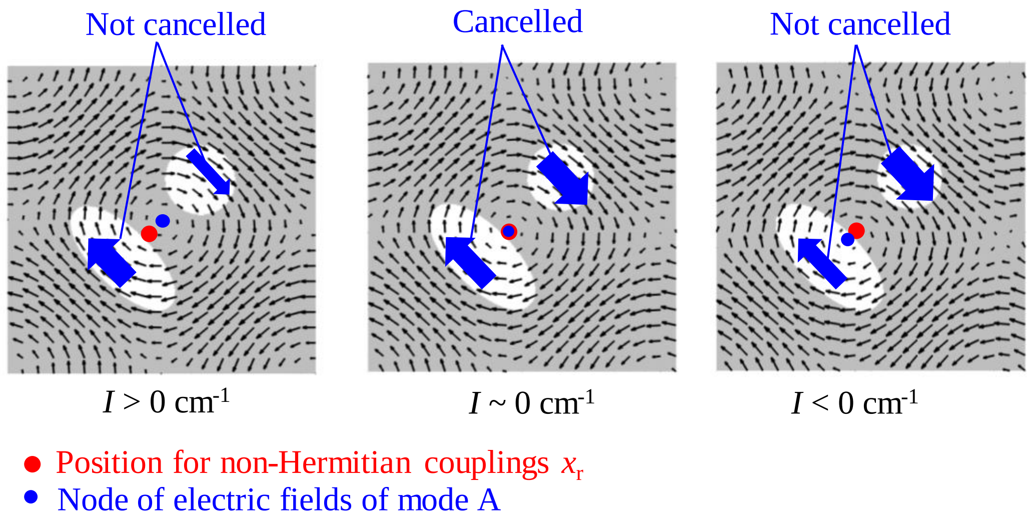

Figure S3| Electric field distributions of mode A in double-lattice photonic crystals with different values of *I*. Arrows indicate the electric field vectors. The red dot indicates the position at which non-Hermitian couplings occur, while the blue dot indicates the node of the electric field distribution.

5. Mode spectrum of a finite-sized PCSEL

In this section, we show an exemplary calculation of the mode frequencies and threshold gains (i.e., mode spectrum) of a finite-sized double-lattice PCSEL, and we demonstrate that the threshold gain margin between the fundamental mode and the higher-order modes originating from band-edge A corresponds to the "global" threshold gain margin, which considers the modes of all band edges, for single-mode lasing in large-area devices. We also discuss the symmetry of the electric field distributions of each mode in the finite-sized PCSEL.

Figure S4a shows the mode spectrum of the 3-mm-diameter double-lattice

PCSEL, which was designed for the simulation in Fig. 6 in the main text (R=45 cm^{-1}, μ=44 cm^{-1}, I=32 cm^{-1}). From this figure, it is obvious that the modes originating from band-edges B, C, and D have much higher total loss than those originating from band-edge A; most of this difference is attributed to the much higher radiation constants of the modes of band-edges B-D, as explained in Supplementary Section 3 (note that the contribution of in-plane loss to this difference is much smaller, owing to the large area of the device). On the other hand, the difference in total loss between the fundamental mode (A-1) and higher-order modes (A-2, A-3) of band-edge A in a 3-mm-diameter PCSEL is much smaller than that between the bands, as shown in the inset. Therefore, it can be concluded that the threshold gain margin between the fundamental mode and the higher-order modes of band-edge A is also the global threshold margin of the device.

Figure S4b shows the electric-field distributions of the modes with the smallest to fifth-smallest total loss in the same device. Here, we consider a photonic crystal which has reflection symmetry along y=x (Γ-M direction), and we assume that the shape of the current injection region is circular, which also maintains the reflection symmetry along y=x. As a result, the electric-field distribution of all modes in the finite-sized PCSEL become either symmetric or anti-symmetric along y=x. Specifically, the higher-order modes that have the lowest and second-lowest total loss (A-2 and A-3) have two antinodes in the Γ-M and Γ-M' directions, so their radiation constants can be described by simply

considering Δk along the Γ-M and Γ-M' directions. Other higher-order modes (A-4, A-5) have more complex electric-field distributions, but their threshold gains are much larger. Therefore, in the discussion of the single-mode stability of PCSELs with reflection symmetry along y=x, it is sufficient to consider the wavenumber dependence of the radiation constants in the Γ-M and Γ-M' directions, which are considered in the main text.

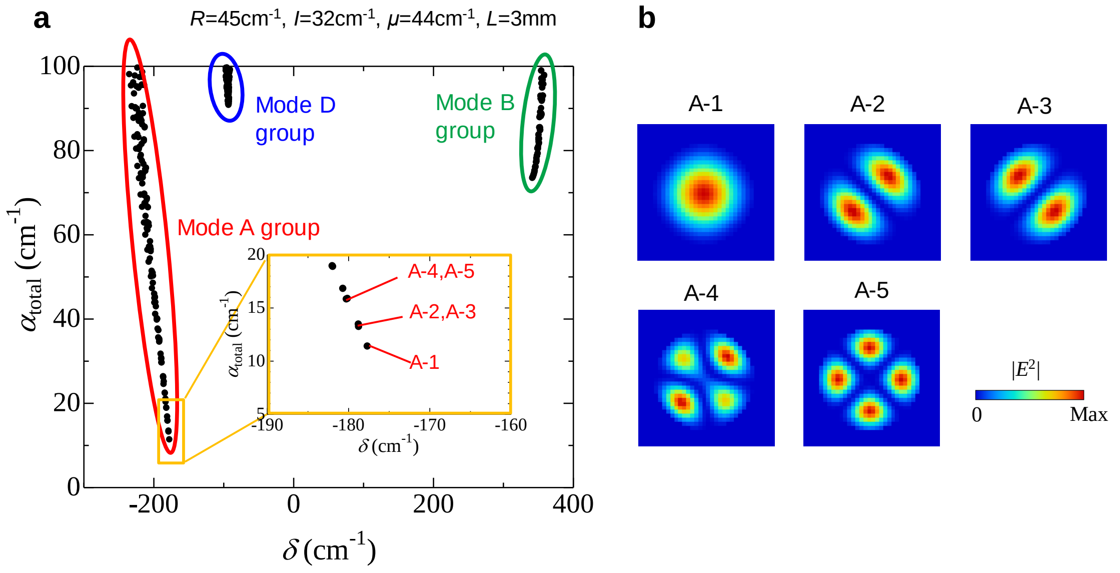

Fig. S4| Calculated total loss and electric-field distributions of eigenmodes in a finite-sized PCSEL (L=3 mm, R=45 cm^{-1}, μ=44 cm^{-1}, I=32 cm^{-1}).

6. Comparison between analytical formulae and numerical simulation of threshold margins of finite-size PCSELs

To verify the analytical formula of the radiation constant difference [Eq. (18)], we numerically calculate the threshold margins between the fundamental mode and first

higher-order mode of 3-mm-diameter PCSELs by using the 3D-CWT for finite-size PCSELs [S2]. In one exemplary simulation, we change the real part of κ_{1D} and fix the other parameters as follows: μ=70 cm^{-1}, Im (κ_{1D}) = 70 cm^{-1}, κ_{2D-} = −70−70i cm^{-1}, κ_{2D+} = −100 cm^{-1}, θ_{pc} = 0.92π. To calculate the in-plane loss of the lasing modes, we assume that a material absorption coefficient of 600 cm^{-1} exists outside the 3-mm-diameter electrode. The simulated results of the radiation constant difference $\Delta\alpha_v$, in-plane loss difference $\Delta\alpha_{//}$, and threshold margin $\Delta\alpha_{total}$ (=$\Delta\alpha_v$+$\Delta\alpha_{//}$) between the fundamental mode and the first higher-order mode are shown in Fig. S5 with red, grey, and black lines, respectively. As is apparent from this result, the radiation constant difference $\Delta\alpha_v$ is much larger than the in-plane loss difference $\Delta\alpha_{//}$ for 3-mm-diameter PCSELs. For comparison, theoretical values of $\Delta\alpha_v$ in Eq. (18) at L=3 mm are shown with a blue line in the same figure. Here, the theoretical result shows excellent agreement with the numerical result (red line), which validates our theory.

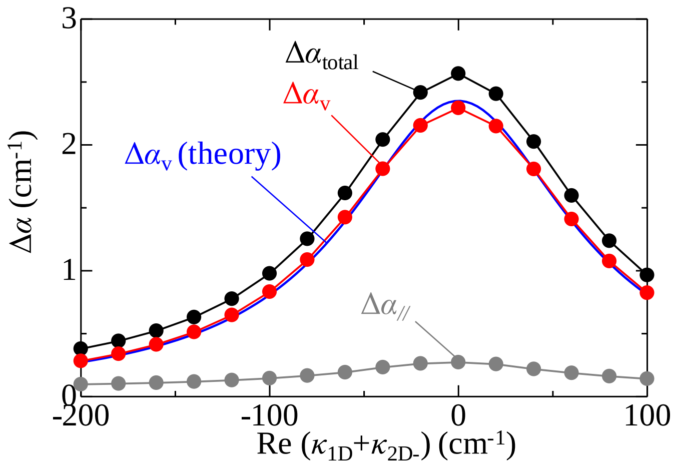

Figure S5| Numerical simulations of radiation constant difference $\Delta\alpha_v$, in-plane loss difference $\Delta\alpha_{//}$, and threshold margin $\Delta\alpha_{total}$ (=$\Delta\alpha_v$+$\Delta\alpha_{//}$) between the fundamental mode and the first higher-order mode in a 3-mm-diameter PCSEL. The blue line shows the theoretical value of α_v in Eq. (18).

7. Fourier component of double-lattice photonic crystals

As explained in the main text, the most dominant Fourier component that determines $\kappa_{1D}+\kappa_{2D-}$ is $\xi_{2,0}$, which induces the direct coupling between R_x and S_x (R_y and S_y). According to Eq. (S8), $\xi_{2,0}$ in a double-lattice photonic crystal composed of an elliptical and circular holes with a lattice separation of d is expressed as

$$\begin{aligned}\xi_{2,0}^{\text{double}} &= \frac{1}{a^2}\int_{\text{unitcell}}\left[n_{\text{ellipse}}^2\left(x+\frac{d}{2},y+\frac{d}{2}\right)+n_{\text{circle}}^2\left(x-\frac{d}{2},y-\frac{d}{2}\right)\right]e^{i(2\beta_0 x)}dxdy \\ &= \xi_{2,0}^{\text{ellipse}}\exp\left(i\frac{4\pi}{a}\cdot\left(-\frac{d}{2}\right)\right)+\xi_{2,0}^{\text{circle}}\exp\left(i\frac{4\pi}{a}\cdot\left(+\frac{d}{2}\right)\right) \\ &= \left(\xi_{2,0}^{\text{ellipse}}+\xi_{2,0}^{\text{circle}}\right)\cos\left(\frac{2\pi}{a}d\right)-i\left(\xi_{2,0}^{\text{ellipse}}-\xi_{2,0}^{\text{circle}}\right)\sin\left(\frac{2\pi}{a}d\right)\end{aligned} \quad , \quad \text{(S22)}$$

where $\xi_{2,0}^{\text{ellipse}}$ and $\xi_{2,0}^{\text{circle}}$ are the Fourier component of the elliptical and circular holes when the center of the hole is placed at the origin. When the filling factors (FF) of these two holes are relatively small, $\xi_{2,0}^{\text{ellipse}}$ and $\xi_{2,0}^{\text{circle}}$ can be approximated as a product of the permittivity difference between GaAs and air $\left(n_{\text{air}}^2-n_{\text{GaAs}}^2\right)$ and the filling factor;

$$\xi_{2,0}^{\text{ellipse}} = \frac{1}{a^2}\int_{\text{unitcell}} n_{\text{ellipse}}^2(\boldsymbol{r})e^{i(2\beta_0 x)}dxdy \sim \left(n_{\text{air}}^2 - n_{\text{GaAs}}^2\right)\cdot FF_{\text{ellipse}}$$
$$\xi_{2,0}^{\text{circle}} = \frac{1}{a^2}\int_{\text{unitcell}} n_{\text{circle}}^2(\boldsymbol{r})e^{i(2\beta_0 x)}dxdy \sim \left(n_{\text{air}}^2 - n_{\text{GaAs}}^2\right)\cdot FF_{\text{circle}} \quad . \qquad \text{(S23)}$$

Substituting Eq. (S23) into Eq. (S22) and assuming d=0.25a+Δd (where Δd is small), we obtain

$$\begin{aligned}\xi_{2,0}^{\text{double}} &= \left(n_{\text{air}}^2 - n_{\text{GaAs}}^2\right)\left[-\left(FF_{\text{ellipse}} + FF_{\text{circle}}\right)\sin\left(\frac{2\pi}{a}\Delta d\right) - i\left(FF_{\text{ellipse}} - FF_{\text{circle}}\right)\cos\left(\frac{2\pi}{a}\Delta d\right)\right] \\ &\simeq \left(n_{\text{GaAs}}^2 - n_{\text{air}}^2\right)\left[\left(FF_{\text{total}}\right)\frac{2\pi}{a}\Delta d + i\left(\Delta FF\right)\right]\end{aligned} \; . \qquad \text{(S24)}$$

8. Models of numerical simulations

Here, we explain the models of the numerical simulations shown in Figs. 5 and 6. The detailed vertical structure of the designed PCSELs is shown in Table S1. The 3D shape of the double-lattice holes was reconstructed from scanning electron microscope images of the cross-section of the holes in the previously fabricated devices, and then was transformed to vary the lattice separation d (0.260a ~ 0.284a) and hole sizes 2x (1.5 nm ~ 6.0 nm). The other parameters were fixed as follows: a = 278 nm, 2l = 125 nm, 2s = 53 nm, 2r = 67 nm. In the simulation of the Hermitian and non-Hermitian coupling coefficients of the real device in Fig. 5, we first calculated the frequencies and radiation constants of the four band-edge modes of the designed PCSELs by rigorous coupled-wave analysis (RCWA) [S3,S4], and then extracted the coupling coefficients by using Eqs. (9) and (10) in the main text. It should be noted that RCWA is more rigorous than

3D-CWT in that it accurately considers all of the mutual couplings of waves including the coupling of TE bands to TM bands, which are not considered in 3D-CWT.

Table S1| Structural parameters of the PCSEL.

Layer	Thickness (nm)	Refractive index
n-clad (AlGaAs)	1000	3.32
AlGaAs	80	3.45
Active (InGaAs/AlGaAs)	(10/20)×3	3.58/3.45
AlGaAs	25	3.27
GaAs	90	3.55
Double-lattice PC	160	n_{pc}
p-clad (AlGaAs)	930~1030	3.32
DBR($Al_xGa_{1-x}As/Al_yGa_{1-y}As$)	(68/78) ×14	3.47/3.01

In the simulation of the time-domain 3D-CWT [S5], we consider not only the mutual couplings of waves but also the carrier-photon interactions such as the carrier-induced changes of the refractive index and gain. The rate equations for the complex amplitude vector $(R_x, S_x, R_y, S_y)^T$ and the carrier density N are expressed as follows;

$$\begin{aligned}\frac{\partial}{\partial t}\begin{pmatrix}R_x\\S_x\\R_y\\S_y\end{pmatrix} &= \frac{c}{n_g}\left[-i\frac{2\pi}{\lambda}\Gamma\Delta n(N)+\frac{\Gamma g(N)-\alpha_{in}}{2}\right]\begin{pmatrix}R_x\\S_x\\R_y\\S_y\end{pmatrix}\\ &+\frac{ic}{n_g}\left(\mathbf{C}_{\text{Hermitian}}+\mathbf{C}_{\text{non-Hermitian}}\right)\begin{pmatrix}R_x\\S_x\\R_y\\S_y\end{pmatrix}-\frac{c}{n_g}\begin{pmatrix}\partial R_x/\partial x\\-\partial S_x/\partial x\\\partial R_y/\partial y\\-\partial S_y/\partial y\end{pmatrix}+\begin{pmatrix}f_1\\f_2\\f_3\\f_4\end{pmatrix}\end{aligned}, \qquad \text{(S25)}$$

$$\frac{\partial N}{\partial t} = \frac{J}{ed_{\mathrm{QW}}} - \frac{N}{\tau_{\mathrm{c}}} - \frac{c}{n_{\mathrm{g}}} g(N)U + D\nabla^2 N \,. \tag{S26}$$

Here, n_{g} is a group refractive index of the multi-layered structure, Γ is an optical confinement factor inside the active layer, $\Delta n(N)$ and $g(N)$ are the carrier-dependent refractive index change and optical gain, respectively, α_{in} is an internal material loss, f_i (i=1–4) is a spontaneous emission term, J is the current density, d_{QW} is the thickness of the active layer, τ_{c} is the carrier lifetime, U is the photon density, and D is the carrier diffusion coefficient. In Eqs. (S25) and (S26), both the electric field vectors and the carrier density (and the other related parameters) depend on the position **r**. The first, second, and third term of the right side of Eq. (S25) denotes carrier-induced frequency and gain change, Hermitian and non-Hermitian mutual couplings, and spatial propagation (including the effect of $\mathbf{C}_{\text{non-Gamma}}$), respectively. In the simulation, we assume a current density distibution $J(\mathbf{r})$ in which the current density at the center of the electrode is 20% less than that at the edge (it should be noted that the current distribution can be controlled by changing the shape of the n-type and p-type electrodes in the experiment). The gain of the active layer $g(N)$ is approximated by using the following linear fractional function of the carrier density with gain saturation, where the effect of gain suppression (compression) due to spectral hole burning and carrier heating is also considered [S6–S8]:

$$g(N)=\frac{g_{\max}\left(N-N_{\mathrm{tr}}\right)}{N+\left[g_{\max}/\left(-g_0\right)\right]N_{\mathrm{tr}}}\cdot\frac{1}{1+\varepsilon U}. \tag{S27}$$

Here, N_{tr} is the transparency carrier density, $g_{\max}$ is the maximum gain, $(-g_0)$ is the absorption coefficient when there are no carriers, and ε is the gain suppression factor considering spectral hole burning and carrier heating. The detailed parameters used for the simulation are summarized in Table S2.

Table S2| Parameters used for 3D-CWT simulations

**Symbol**	**Parameter**	**Value**
a	Lattice constant	278 nm
d_{QW}	Thickness of InGaAs wells	30 nm
n_{g}	Group refractive index	3.481
n_{eff}	Effective refractive index	3.411
Γ	Optical confinement factor	0.064
$g_{\max}$	Maximum gain	2000 cm^{-1}
g_0	Absorption coefficient	−5000 cm^{-1}
N_{tr}	Transparency carrier density	$1.5\times10^{18}\ \mathrm{cm}^{-3}$
ε	Gain suppression factor	$2.45\times10^{-17}\ \mathrm{cm}^{3}$
$\mathrm{d}n/\mathrm{d}N$	Refractive index change coefficient	$-7.6\times10^{-21}\ \mathrm{cm}^{3}$
D	Diffusion constant	100 cm^2/s
α_{in}	Internal material loss	3.0 cm^{-1}
τ_{c}	Carrier lifetime	2 ns

9. Carrier-induced radiation constant change and frequency change

From Eq. (16), we obtain

$$\left[\delta'_{\mathrm{A}}+i\left(\alpha_{\mathrm{A}}/2-\mu\right)\right]^2=\left(R+i\mu\right)^2+I^2+\left(\Delta k/\sqrt{2}\right)^2,\quad \delta'_{\mathrm{A}}=\delta_{\mathrm{A}}-\kappa_{11}-\kappa_{2\mathrm{D}+}\,. \tag{S28}$$

Taking the imaginary part of both sides in Eq. (S28), we obtain

$$\delta'_{\mathrm{A}}\left(\alpha_{\mathrm{A}}-2\mu\right)=2R\mu\,. \tag{S29}$$

It should be noted that the right hand side of Eq. (S29) is constant. Considering the carrier-induced small change in Eq. (S29), we obtain

$$\left(\Delta\delta'_{\mathrm{A}}/\Delta N\right)\left(\alpha_{\mathrm{A}}-2\mu\right)+\delta'_{\mathrm{A}}\left(\Delta\alpha_{\mathrm{A}}/\Delta N\right)=0\,. \tag{S30}$$

$$\frac{\Delta\alpha_{\mathrm{A}}/\Delta N}{\Delta\delta'_{\mathrm{A}}/\Delta N}=\frac{2\mu-\alpha_{\mathrm{A}}}{\delta'_{\mathrm{A}}}\,. \tag{S31}$$

Eq. (21) in the main text is directly obtained from Eq. (S31) in the vicinity of the Γ point ($\delta'_{\mathrm{A}}\sim -R$, $\alpha_{\mathrm{A}}\ll\mu$).

10. Potential challenges and solutions for experimentally realizing 100-W-to-1-kW PCSELs

To experimentally realize the 100-W-to-1-kW PCSELs discussed in the main text, it is important to control the current density distribution of the device as well as to dissipate a large amount of heat generated inside of the device. Concerning the former, it is difficult to inject current into the center of a large-area device using a conventional ring-window

electrode; to realize a spatially uniform current distribution, a mesh-type electrode, as was investigated in Ref. S9 for example, must be employed instead. In the case of a 3-mm-diameter PCSEL, by employing the mesh-type electrode shown in Fig. S6a, we can realize the current density distribution shown in Fig. S6b, in which the current density at the center of the electrode is only 20% lower than that at the edge. This current distribution is almost equal to that assumed in our time-domain simulations. It should be noted that we have numerically confirmed that the impact of the mesh on the beam profile is small because the width of each mesh (15 μm) is two orders of magnitude smaller than the diameter of the device (3 mm). In addition, the employment of the mesh-type electrode also contributes to the reduction of the series resistance of the device, which is useful to decrease the heat generated inside the device, which is discussed in the next paragraph. A similarly designed mesh-type electrode can be also applied to 10-mm-diameter PCSELs.

As for thermal management, the amount of heat generated inside of the device is negligible in the case of short-pulse operation with pulse widths of less than 1 μs, which is used for LiDAR and micro-processing. For continuous-wave (CW) operation, however, it is necessary to mount the device to a cooling package for heat dissipation. In this case, since the maximum heat dissipation per unit area is fixed, the maximum CW emission power is nearly proportional to the device area. Based on a CW emission power of ~7W

achieved using an 800-μm-diameter PCSEL [S10], it is expected that CW emission powers of ~98 W and ~1090 W are achievable using 3-mm-diameter and 10-mm-diameter PCSELs, respectively. For example, we have calculated that, for a 3-mm-diameter PCSEL mounted to a water-cooling package at 20°C as shown in Fig. S6c, we can suppress the maximum temperature of the device to below 60°C even under a heat generation of 200 W (which corresponds to an injection current of ~130 A and a CW output power of ~100 W) as shown in Fig. S6d. It should be also noted that the non-uniform temperature distribution shown in Fig. S6d and the resultant band-edge frequency distribution may affect the lasing characteristics of the device, but the effects of these distributions can be compensated by spatially varying the lattice constant of the photonic-crystal structure [S11]. Therefore, the 100-W-to-1-kW-class PCSELs proposed in this paper are experimentally feasible.

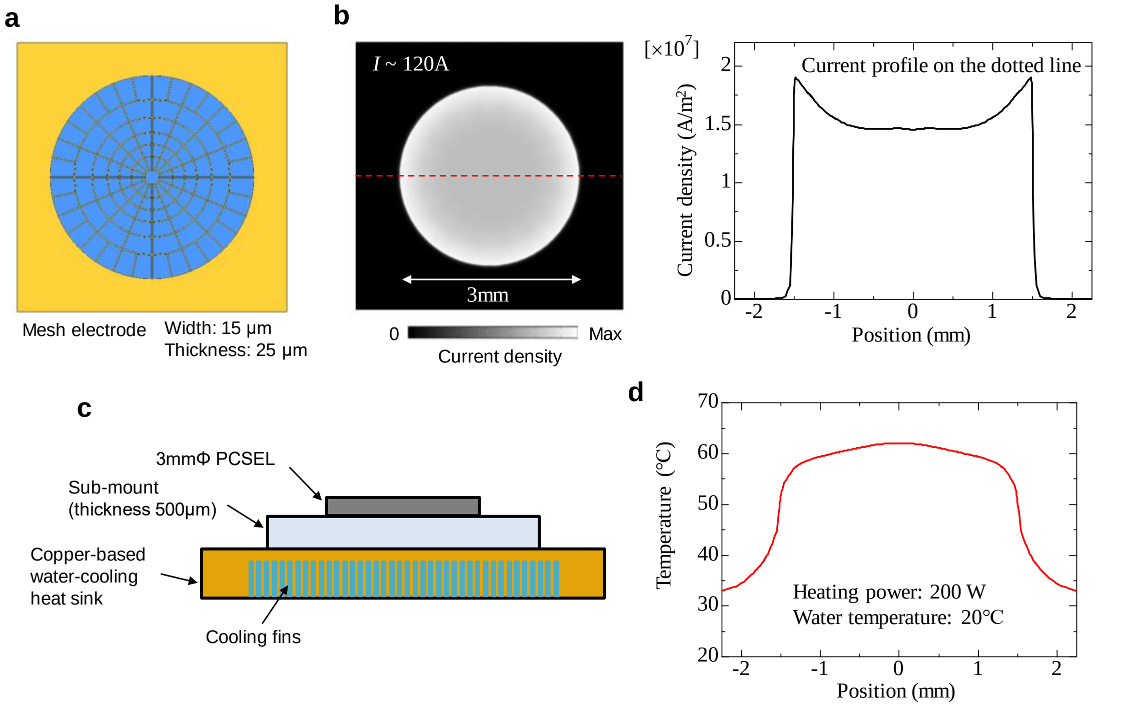

Fig. S6| Control of current density distribution and temperature distribution in a large-area PCSEL. a, Schematic of a mesh-type electrode on a large-area PCSEL. **b,** Calculated current density distribution of the device shown in **a**. **c,** Schematic of mounting of a large-area PCSEL to a sub-mount and a heat sink. **d,** Calculated temperature distribution of the device shown in **c**.

Supplementary References

[S1] Liang, Y., Peng, C., Sakai, K., Iwahashi, S. & Noda, S. Three-dimensional coupled-wave model for square-lattice photonic crystal lasers with transverse electric polarization: a general approach. *Phys. Rev. B* **84**, 195119 (2011).

[S2] Liang, Y., Peng, C., Sakai, K., Iwahashi, S. & Noda, S. Three-dimensional coupled-wave model for square-lattice photonic crystal lasers with transverse electric polarization: finite-size effects. *Opt. Express* **20**, 15945–15961 (2012).

[S3] Lin, L.-K., Li, Z.-Y. & Ho, K.-M. Lattice symmetry applied in transfer-matrix methods for photonic crystals. *J. Appl. Phys.* **94**, 811–821 (2003).

[S4] Song, A. Y., Kalapala, A. R. K., Zhou, W. & Fan, S. First-principles simulation of photonic crystal surface-emitting lasers using rigorous coupled wave analysis. *Appl. Phys. Lett.* **113**, 041106 (2018).

[S5] Inoue, T. *et al.* Comprehensive analysis of photonic-crystal surface-emitting lasers via time-dependent three-dimensional coupled-wave theory. *Phys. Rev. B* **99**, 035308 (2019).

[S6] Chen, S., Yoshita, M., Ito, T., Mochizuki, T., Akiyama, H. & Yokoyama, H. Gain-switched pulses from InGaAs ridge-quantum-well lasers limited by intrinsic dynamical gain suppression. *Opt. express* **21**, 7570–7576 (2013).

[S7] Huang, J. & Casperson, L. W. Gain and saturation in semiconductor lasers. *Opt. and quantum electron.* **25**, 369–390 (1993).

[S8] Willatzen, W., Takahashi, T. & Arakawa, Y. Nonlinear gain effects due to carrier heating and spectral holeburning in strained-quantum well lasers. *IEEE Trans. Photon. Technol. Lett.* **4**, 682–685 (1992).

[S9] Wang, Z. *et al*, Large area photonic crystal quantum cascade laser with 5 W surface-emitting power, *Opt. Express* **27**, 22708–22716 (2019).

[S10] Zoysa, M. D. *et al*, Thermal management for CW operation of large-area double-lattice photonic-crystal lasers, *J. Opt. Soc. Am B* **37**, 3882–3887 (2020).

[S11] Katsuno, S. *et al*, Self-consistent analysis of photonic-crystal surface-emitting lasers under continuous-wave operation, *Opt. Express* **29**, 25118–25132 (2021).
